# Three Mitochondrial Genomes of Chrysochroinae (Coleoptera, Buprestidae) and Phylogenetic Analyses

**DOI:** 10.3390/genes15101336

**Published:** 2024-10-17

**Authors:** Bowen Ouyang, Xuyan Huang, Yujie Gan, Zhonghua Wei, Aimin Shi

**Affiliations:** 1 College of Life Sciences, China West Normal University, Nanchong 637009, China; oybw18990894948@163.com (B.O.); huangxy9999@gmail.com (X.H.); gyj999@foxmail.com (Y.G.); aiminshi@cwnu.edu.cn (A.S.); 2 The Key Laboratory of Southwest China Wildlife Resources Conservation of the Ministry of Education, College of Life Sciences, China West Normal University, Nanchong 637009, China

**Keywords:** *Catoxantha*, Chrysochroinae, jewel beetles, mitogenome, new record, *Nipponobuprestis*, phylogeny

## Abstract

Three mitochondrial genomes of Chrysochroinae (Buprestidae) were sequenced and analyzed. The mitogenomes of the genera *Catoxantha* and *Nipponobuprestis* are first reportedand *Chrysochroa opulenta* is a first record for China. The complete mitogenomes of *Catoxantha luodiana*, *Nipponobuprestis guangxiensis* and *Chrysochroa opulenta* exhibit striking similarities in their lengths and composition. Specifically, their lengths are 15,594 bp, 15,775 bp and 15,587 bp, respectively. Each of these genomes encodes 37 typical mitochondrial genes. The overwhelming majority of protein-coding genes (PCGs) have the typical ATN (ATT, ATA, ATG or ATC) as the start codon and terminate with TAR (TAA or TAG) as the stop codon or an incomplete stop codon T-. Among the three mitogenomes, Leu2, Ser2 and Phe were the most frequently encoded amino acids. In the PCGs, the Ka/Ks ratio of *cox1* is the lowest, whereas *atp6* has the highest value. This suggests that *cox1* can be used as a molecular barcode for species delimitation and phylogeny in Chrysochroinae. The phylogenetic results showed that *C*. *luodiana* and two *Chrysochroa* species formed a clade. Based on the topology of the phylogenetic tree, the genus *Catoxantha* should be reassigned as a subgenus of *Chrysochroa*.

## 1. Introduction

The buprestid subfamily Chrysochroinae Laport includes eight tribes, distributed worldwide, especially in tropical and subtropical regions. Many specimens of Chrysochroinae have been treated as collectibles and decorations because most species have large bodies and conspicuous colors. However, there have been a few biological studies of Chrysochroinae, including developmental, life history and behavioral studies [1,2,3,4]. The overwintering habitat of *Dicerca asperata* was reported by Karns and Behrendt [5]. Antennal responses of *Ovalisia festiva* (Linnaeus, 1767) to volatile compounds of *Thuja occidentalis* Linnaeus, 1753 were studied by Bozsik et al. [6].

Studies on the phylogeny of Chrysochroinae are few and the interrelationships within Chrysochroinae are not well resolved. Hołyński [7] used morphological characteristics to explore the interrelationships of Chrysochroinae. The mitochondrial genes *COI* and *16S* were used for species identification of *Chrysochroa* Dejean, 1833 [8]. Based on molecular data, phylogenetic studies suggest that Chrysochroinae is close to Buprestinae; however, systematic problems have not been thoroughly addressed [9,10]. In recent years, some buprestid mitogenomes have been reported but only four mitogenomes are currently known for Chrysochroinae [10,11,12,13].

In this study, the complete mitogenomes of *Catoxantha luodiana* [14], *Nipponobuprestis guangxiensis* and *Chrysochroa opulenta* were sequenced and annotated [5,15]. Their composition and structure were also analyzed. The mitogenomes of the genera *Catoxantha* Dejean, 1833 and *Nipponobuprestis* Obenberger, 1942 are first reported and the occurrence of *Chrysochroa opulenta* is a first record for Yunnan Province, China. To explore the phylogenetic relationships of these genera, phylogenetic trees were constructed using concatenated sequences of protein-coding genes (PCGs) and ribosomal RNA genes (rRNAs).

## 2. Material and Methods

### 2.1. Taxon Sampling and DNA Extraction

The specimens of *Catoxantha luodiana* and *Nipponobuprestis guangxiensis* were collected in Yachang Towns, Leye County, Guangxi Zhuang Autonomous Region, China, on 21 April 2021 and 26 May 2022, respectively. The specimens of *Chrysochroa opulenta* were collected in Nongzhang Town, Yingjiang County, Yunnan Province, China, on 15 April 2023. All the specimens were identified based on morphological descriptions and illustrations [7,14,15]. The specimens were preserved in 95% ethanol at −24 °C in the China West Normal University. The muscle tissue of the legs was used for genomic DNA extraction using the DNeasy Blood and Tissue Kit Purification Kit (Shanghai, China). In this study, the experimental procedure is carried out according to the operating instructions.

### 2.2. Mitogenome Sequencing, Annotation and Analyses

The raw data of mitogenomes were sequenced by next-generation sequencing. A library was constructed using the whole genome shotgun method (150 bp paired-end reads) based on the Illumina MiSeq platform. The average sequencing coverage of *C. luodiana*, *N. guangxiensis* and *Ch. opulenta* is 245.6×, 252.2× and 236.8×, respectively. The low sequences of raw data were removed using Trimmomatic v. 0.36 [16]. The clean data were used for assembly using Geneious v. 11.0.2 [17]. The mitochondrial genes were annotated on the MITOS web server (http://mitos.bioinf.uni-leipzig.de/index.py, accessed on 27 September 2024) with the invertebrate mitotic gene code [18]. By comparing homologous genes with closely related species, the start and end sites of 13 protein-coding genes (PCGs) were manually corrected. The positions of two ribosomal RNA genes (rRNAs) were also determined by comparing them with homologous genes of closely related species. The predicted secondary structures of transfer RNA genes (tRNAs) were visualized using VARNA v. 3.9 [19]. The nucleotide composition and the strand asymmetry of mitogenomic sequences [20] and the relative synonymous codon usage (RSCU) and of PCGs were analyzed with MEGA v. 12 [21]. The synonymous substitutions (Ks), non-synonymous substitutions (Ka) and rate of Ka/Ks for PCGs were calculated using DnaSP v. 5.0 [22]. These three new mitogenomic sequences are available on GenBank (*C. luodiana* accession no. PP211020; *N. guangxiensis* accession no. PP133641; *Ch. opulenta* accession no. PP211021).

### 2.3. Phylogenetic Analyses of Buprestidae

A total of 30 buprestid species, representing five subfamilies, were used for phylogenetic analyses, including the three complete mitogenomic sequences provided in this study (Table 1). *Dryops ernesti* Gozis, 1866 and *Heterocerus parallelus* Gebler, 1830 were used as outgroups [23]. The sequences of PCGs and rRNAs were aligned using MAFFT v. 7.0 [24]. The aligned sequences were trimmed and concatenated using trimAl v. 1.2 and the ‘Concatenate Sequence’ tool plugged into PhyloSuite v. 1.2.2 [25,26]. ModelFinder was used to calculate the suitable model for phylogenetic analyses [27]. The most suitable models GTR+I+G for 13 PCGs + 2 RNAs were used for maximum likelihood (ML) analyses and Bayesian analyses using IQ-TREE v. 1.6.8 and MrBayes v. 3.2.7 [28,29], respectively. The original phylogenetic trees were edited using Figtree v. 1.43. For ML analyses, parameters were used as follows: number of bootstraps: 5000, replicates: 1000, minimum correlation coefficient 0.9. For MrBayes analyses, parameters were used as follows: generations: 2,000,000, sampling frequency: 100, number of runs: 2, burn-in fraction: 0.25. The Markov chain Monte Carlo chains were executed independently following the exclusion of constant sites from the alignment, and they were terminated once satisfactory convergence was achieved in both runs. A consensus tree was generated from the remaining trees after eliminating the first 25% of trees as burn-in.

## 3. Results

### 3.1. Genome Organization and Base Composition

The complete mitogenomes of the buprestid species *C. luodiana* (GenBank No. PP211020), *N. guangxiensis* (No. PP133641) and *Ch. opulenta* (No. PP211021) were sequenced and annotated in this study. These three newly sequenced mitogenomes have the same composition, consisting of 37 coding genes (13 PCGs, 22 tRNAs and two rRNAs) and a control region (A+T-rich region). Among them, four PCGs (*nad1*, *nad4*, *nad4L* and *nad5*), eight tRNAs (*trnC*, *trnF*, *trnH*, *trnL1*, *trnP*, *trnQ*, *trnV* and *trnY*) and two rRNAs are encoded on the N-strand, while the other 23 genes (14 tRNAs and 9 PCGs) are encoded on the J-strand (Figures S1–S3), which agrees with previous studies [10,11,12,23,30,31,32,33,34,35,36,37,38].

Three new mitogenome sequences had a high A+T content (67.22–69.71%), high AT-skews and low GC-skews. The AT-skews were 0.12 (*C. luodiana*), 0.14 (*N. guangxiensis*) and 0.16 (*Ch. opulenta*) (Table 1). *C. luodiana* has 15 overlapping regions with a total length of 37 bp, *N. guangxiensis* has 10 overlapping regions with a total length of 31 bp and *Ch. opulenta* has 13 overlapping regions with a total length of 35 bp (Table 2). *C. luodiana* has 6 spacer regions, *N. guangxiensis* has 7 spacer regions and *Ch. opulenta* has the longest spacer region of up to 12 regions. Meanwhile, *Ch. opulenta* has a spacer of 85 bp between *rrnL* and *trnA*, which is the longest spacer region among the three species. The largest overlap is between the *trnD* and *atp8* genes whose lengths are 36 bp (*C. luodiana*), 52 bp (*N. guangxiensis*) and 17 bp (*Ch. opulenta*). The nucleotide composition, codon usage and gene order of these three new mitogenomes were consistent with other known Buprestidae species [10,11,12,23,30,31,32,33,34,35,36,37,38]. Gene rearrangement was not found in these three species.

### 3.2. Protein-Coding Regions, Codon Usage and Nucleotide Diversity

The lengths of the protein-coding genes (PCGs) in the genomes of these three species, 11,128 bp (*C. luodiana*), 11,158 bp (*N. guangxiensis*) and 11,154 bp (*Ch. opulenta*), accounted for between 70.73% and 71.56% of the mitogenome. The mitogenomes have the potential to encode 3699 to 3709 amino acid-coding codons, excluding stop codons. In the PCGs, *nad5* is the largest gene ranging from 1714 bp to 1720 bp, while *atp8* is the smallest gene ranging from 156 bp to 180 bp, which is consistent with the other buprestid mitogenomes [10,11,12,23,30,31,32,33,34,35,36,37,38]. Most PCGs typically initiate translation using ATN (ATA/ATT/ATG/ATC) as the start codon, with the *nad1* gene being an exception as it starts with TGG or TTG [10,30]. The less common *nad1* start codon has also been found in the mitogenomes of other insects [31,39]. The start codon of *cox1* (*C. luodiana*, *N. guangxiensis*) is indeterminate and it may have an unusual start codon [39,40,41,42,43]. Stop codons of nine PCGs are complete TAA or TAG, while stop codons of four PCGs are incomplete T-. The incomplete stop codon T is generated by adding 3′ residues of A to the mRNA [44,45].

**Figure 1:**
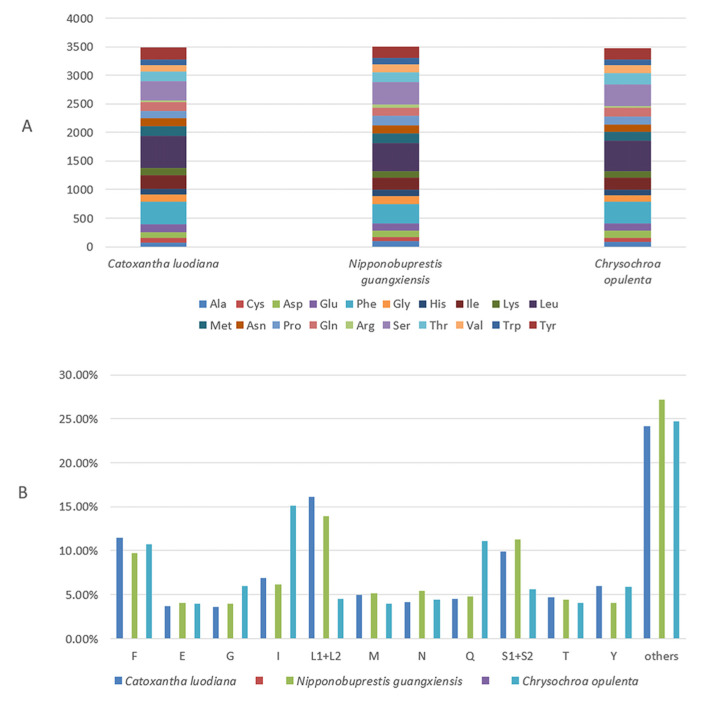
Numbers of different amino acids in the three new mitogenome sequences (**A**) and the percentages of the top eleven amino acids (**B**). The stop codon is not included in these graphs.

**Figure 2:**
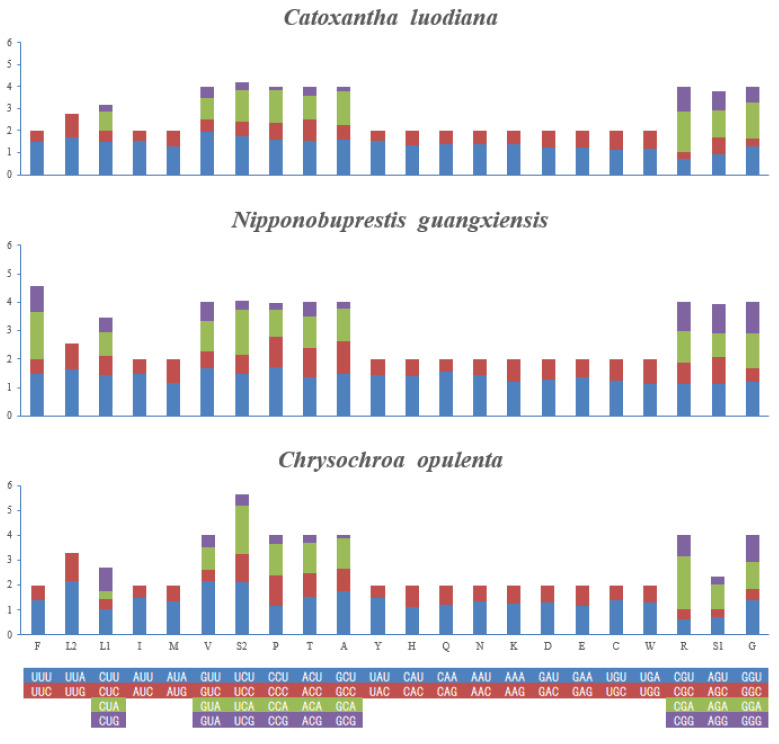
Relative synonymous codon usage (RSCU) of the three new mitogenome sequences.

Numbers of different amino acid sequences and 11 most frequently used amino acid percentages of these three new mitogenomes are provided (Figure 1). Among them, the L1 usage rate was the highest, while the R usage rate was the lowest. The relative synonymous codon usage (RSCU) value indicates that the three most commonly used amino acids are L2, S2 and P (Figure 2, and the most commonly used codons are TTA (L2), TCT (S2) and CCT (P).

**Figure 3:**
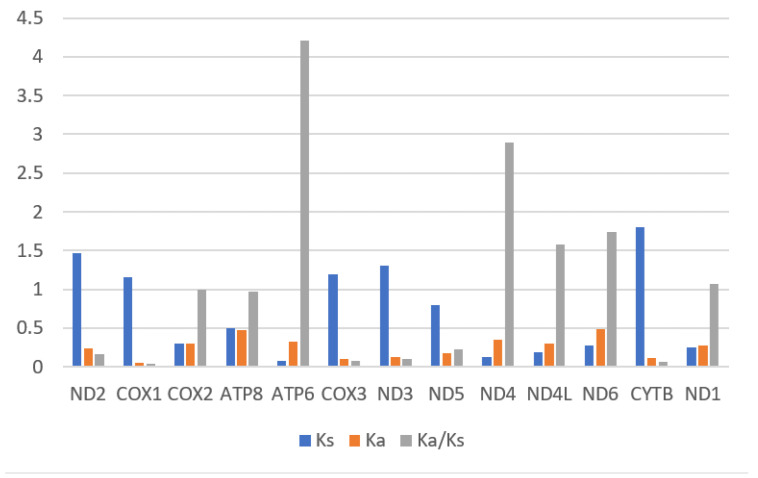
The ratio of Ka/Ks of 13 PCGs in the three newly sequenced buprestid mitogenomes.

The values of Ka, Ks and Ka/Ks (Figure 3) for PCGs of these three mitogenomes showed Ka/Ks representing three different types of selection: positive selection (Ka/Ks > 1), negative selection (Ka/Ks < 1) and neutral selection (Ka/Ks = 1) [46,47]. The lowest value of Ka/Ks was found in *cox1* (0.043), while *atp6* had the highest value (4.2). This suggests that the gene *cox1* is subjected to the highest purification selection [45]. Pi values were calculated for 13 PCGs across the three species, ranging from 0.183 to 0.346. The PCG with the highest variability was *atp8* (Pi = 0.346), while *cox1* (Pi = 0.183) had the lowest variability.

### 3.3. Ribosomal and Transfer RNA Genes

The length of *16S* ranges from 1198 bp (*Ch. opulenta*) to 1301 bp (*C. luodiana*), while the length of *12S* ranges from 623 bp (*Ch. opulenta*) to 771 bp (*N. guangxiensis*). The rRNA genes are situated between *trnL1* and the A+T-rich region, which is separated by *trnV*, in accordance with findings from previous studies in Buprestidae [10,12,23,30,31,32,33,34,35,36,37]. The total length of the 22 tRNA genes varied between 1438 (*Ch. opulenta*) bp and 1448 (*C. luodiana*) bp. Meanwhile, the length of the individual tRNA gene typically ranged from 61 bp to 72 bp. In total, 14 tRNA genes are encoded on the J-strand, and 8 are encoded on the N-strand. All tRNAs exhibit a conventional shamrock-like secondary structure, except for *trnS1,* which lacks the dihydrouridine (DHU) arm [48,49,50,51,52]. The *trnS1* genes of the three species are nearly identical in size, measuring 68, 67 and 67 bp (Figures S4–S6). In some tRNAs, the presence of a U-G mismatch was detected, which also occurs in other buprestid species [10,13,30,31,36].

### 3.4. A+T-Rich Region and Gene Arrangement

The A+T-rich region, commonly referred to as the control region (CR), is widely recognized as the largest non-coding segment within mitogenomes. The total lengths of the A+T-rich regions in three species are 949 bp (*C. luodiana*), 1040 bp (*N. guangxiensis*) and 1050 bp (*Ch. opulenta*). The A+T-rich regions within the genomes of these three eukaryotic species are positioned between *trnI* and *rrnS*. In addition, the A+T content (76.35–78%) in the A+T-rich regions is higher than that of the complete mitogenome (67.22–69.71%), PCGs (65.33–68.68%), rRNAs (70.46–71.92%) and tRNAs (69.96–70.51%) (Table 3).

The gene order of three new mitogenomes is consistent with the others in Buprestidae. The largest intergenic region is 8 bp (*C. luodiana*, *N. guangxiensis* and *Ch. opulenta*) located between *trnW* and *trnC*, while the largest size of gene overlap is 52 bp (*N. guangxiensis*) located between *trnD* and *atp8*. The number of overlapping regions in complete mitogenomes of *C. luodiana*, *N. guangxiensis* and *Ch. opulenta* is 15, 10 and 13, respectively.

### 3.5. Phylogenetic Analyses

**Figure 4:**
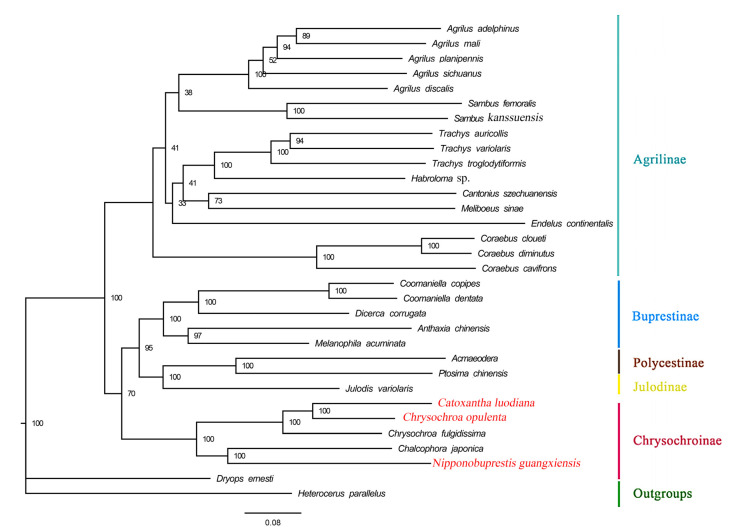
Maximum likelihood tree of 30 Buprestidae species based on 13 PCGs + 2 rRNAs. Values at nodes are bootstrap support values.

**Figure 5:**
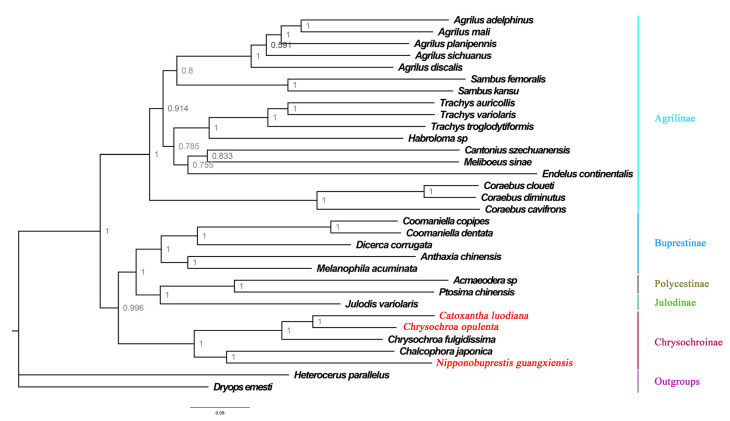
Bayesian tree of 30 Buprestidae species based on 13 PCGs + 2 rRNAs. Values at nodes are posterior probability.

In this study, phylogenetic trees were constructed using a nucleotide dataset (13 PCGs + 2 rRNAs) derived from 33 species, including *Heterocerus parallelus* and *Dryops ernesti* as outgroups. Despite using different methods, the topologies of the phylogenetic trees were generally consistent. All buprestid species are grouped in a large, independent clade (Figure 4 and Figure 5). The subfamily-level relationship was (Chrysochroniae + ((Julodinae + Polycestinae) + Buprestinae) + Agrilinae) (Figure 4 and Figure 5). The genera *Chalcophora* Dejean, 1833, *Chrysochroa*, *Nipponobuprestis* and *Catoxantha* are grouped together and comprise the subfamily Chrysochroniae with high support values (ML: 100; BI: 1). Based on the topologies of this subfamily in the ML and BI trees, the target species *Catoxantha luodiana* is surrounded by *Chrysochroa opulenta* and *Chrysochroa fulgidissima*. This result suggests that *Catoxantha luodiana* is a member of the genus *Chrysochroa*. It also indicates that *Catoxantha* should be relegated to a subgenus of *Chrysochroa*, which supports the classification proposed by Hołyński [7] based on morphological characteristics. Further, the target species *Nipponobuprestis guangxiensis* is the sister species of *Chalcophora japonica* with high support values (ML: 100; BI: 1).

## 4. Discussion

The complete mitogenome can provide far more information than that of fragmented nucleotide sequences and is, therefore, a more valuable genetic marker for studying species delimitation and phylogenetic relationships [36,37]. Thus, the complete mitogenome genomic data of the present study yielded sequence characteristics, gene composition, gene arrangement, codon usage and the phylogenetic position of *C. luodiana*, *N. guangxiensis* and *Ch. opulenta* in Buprestidae.

The results suggested that these three mitogenomes contain 37 typical genes (13 PCGs, 22 tRNAs, 2 rRNAs) and a control region. Compared with previous studies [23,29,30,33], the length of three new complete mitogenomes is medium length, which is mainly caused by the short length of CR. The base composition of mitogenomes of *C. luodiana*, *N. guangxiensis* and *Ch. opulenta* are congruent with those of other Chrysochroinae species [10,12,13]. Compared with most previous studies [11,23,29,31,32,33,37], these three sequences have a lower A + T content and higher AT-skews. The start codon *nad1* (*C. luodiana N. guangxiensis* and *Ch. opulenta*) is TGG, TTG and TTG, while the start codon of *nad4L* and *nad5* (*N. guangxiensis*) is GTG, which has also been found in previous studies [10,29]. The *cox1* gene, with a Pi value of 0.183, exhibited the lowest variability, and the *cox1* had the lowest value of Ka/Ks, which is not exceptional but occurs in almost all insects and even animals [23]. Compared with previous studies [23,29,31,33], the length of tRNAs is also medium length and *trnS1* has a dihydrouridine (DHU) arm. Base–base mismatches are also discovered in arms of tRNAs, which is congruent with other studies of buprestid beetles [10,23,30].

The taxonomic status of the genus *Megaloxantha* Kerremans, 1903 [53] is controversial. This genus was transferred to *Chrysochroa* as a subgenus including nine species, based on a morphological cladistic analysis [7]. Later, the subgenus *Megaloxantha* was treated as a synonym of the genus *Catoxantha* by Kubáň [54]. Although some phylogenetic analyses of Buprestidae have been performed based on morphology [55], genes [9], mitogenomes [10,11,23,29,33], the *Catoxantha*, *Chrysochroa* and *Nipponobuprestis* are not included, except the study of Evans et al. [9]. In the present study, the topologies of phylogenetic trees show that the relationship of each subfamily is different with the previous study [9], which is due to the different datasets used in these two studies: previous study used 4 genes of 141 ingroup species, while this study used mitogenome of 27 ingroup species. The phylogenetic result also showed that *Catoxantha luodiana* is surrounded by *Chrysochroa* species, implying that *Catoxantha* as a subgenus of *Chrysochroa* is more reasonable. The Buprestidae, one of the largest groups within Coleoptera, requires more mitogenomes to better elucidate phylogenetic relationships and taxonomic status of subfamilies, tribes and genera.

## 5. Conclusions

In this study, the complete mitogenome of *C. luodiana*, *N. guxngxiensis* and *Ch. opulenta* were described and analyzed. Among them, the mitogenomes of the genera *Catoxantha* and *Nipponobuprestis* are reported for the first time. The total length of the three mitogenomes ranges from 15,587 bp to 15,775 bp. The gene order in these three mitogenomes agrees with previously known buprestid species. Examination of three mitogenomes indicates a lack of significant differences in both AT content and AT bias. All of them exhibit a positive AT skew and a pronounced AT bias. The initiation codon for all PCGs follows the typical ATN pattern, except for *nad1* in *C. luodiana*, *N. guangxiensis* and *Ch. opulenta*, which is TGG, TTG and TTG, respectively. Additionally, *nad4L* and *nad5* in *N. guangxiensis* commence with the start codon GTG. The results of the phylogenetic analyses suggest that *C. luodiana* is a member of the genus *Chrysochroa*, and the species *N. guangxiensis* is closely related to *Ch. japonica*.

## Figures and Tables

**Table 1:** Information on the buprestid mitogenomes and outgroups used for phylogeny.

Taxa	Accession No.	Genome Size (bp)	A + T%	AT-Skew	References
*Agrilus sichuanus* Jendek, 2011	OK189519	16,521	71.73	0.12	[30]
*Agrilus mali* Matsumura, 1924	MN894890	16,204	74.46	0.08	[31]
*Agrilus planipennis* Fairmaire, 1888	KT363854	15,942	71.90	0.12	[32]
*Agrilus discalis* Saunders, 1873	ON644870	15,784	74.59	0.11	[33]
*Coraebus diminutus* Gebhardt, 1928	OK189521	15,499	68.42	0.12	[30]
*Coraebus cloueti* Théry, 1895	OK189520	15,514	69.27	0.11	[30]
*Coraebus cavifrons* Descarpentries and Villier, 1967	MK913589	15,686	69.79	0.12	[34]
*Meliboeus sinae* Obenberger, 1935	OK189522	16,108	72.42	0.11	[30]
*Sambus femoralis* Kerremans, 1892	OK349489	15,367	73.23	0.12	[30]
*Sambus kanssuensis* Ganglbauer, 1890	OQ784265	15,411	72.4	0.10	[33]
*Habroloma* sp.	OQ784266	16,273	73.99	0.11	[33]
*Endelus continentalis* Obenberger, 1944	OL702762	16,246	75.60	0.13	[33]
*Cantonius szechuanensis* Obenberger, 1958	OQ784264	15,927	73.09	0.11	[33]
*Trachys auricollis* Saunder, 1873	MH638286	16,429	71.05	0.10	[23]
*Trachys troglodytiformis* Obenberger, 1918	KX087357	16,316	74.62	0.10	Unpublished
*Trachys variolaris* Saunders, 1873	MN178497	16,771	72.11	0.11	[35]
*Catoxantha luodiana* (Yang and Xie, 1993)	PP211020	15,594	68.68	0.12	This study
*Anthaxia chinensis* Kerremans, 1989	MW929326	15,881	73.61	0.09	[36]
*Coomaniella dentata* Song, 2021	OL694144	16,179	76.59	0.01	[10]
*Coomaniella copipes* Jendek and Pham, 2013	OL694145	16,196	74.47	0.03	[10]
*Melanophila acuminata* (De Geer, 1774)	MW287594	15,853	75.66	0.02	[37]
*Nipponobuprestis guangxiensis* Peng, 1995	PP133641	15,775	65.33	0.14	This study
*Chalcophora japonica* (Gory, 1840)	OP388137	15,759	67.97	0.13	[11]
*Chrysochroa fulgidissima* Schönherr, 1817	EU826485	15,592	69.92	0.15	[12]
*Dicerca corrugata* Fairmaire, 1902	OL753086	16,276	71.76	0.09	[10]
*Chrysochroa opulenta* (Gory, 1832)	PP211021	15,587	67.16	0.16	This study
*Acmaeodera* sp.	FJ613420	16,217	68.41	0.11	[38]
*Ptosima chinensis* Marseul, 1867	OP388449	16,115	67.00	0.13	[11]
*Julodis variolaris* (Pallas, 1771)	OP390084	16,227	70.43	0.12	[11]
*Dryops ernesti* Gozis, 1886	KX035147	15,672	72.98	0.07	Unpublished
*Heterocerus parallelus* Gebler, 1830	KX087297	15,845	74.03	0.13	Unpublished

**Table 2:** The mitogenomes of *Catoxantha luodiana*, *Nipponobuprestis guangxiensis* and *Chrysochroa opulenta*. ? not determined.

Gene	Strand	Position		Codons		Intergenic Nucleotides
		From	To	Start	Stop	
*trnI*	J	1/1/1	67/66/65			0/0/0
*trnQ*	N	65/64/63	133/132/131			−3/−3/−3
*trnM*	J	133/132/131	201/201/199			−1/−1/−1
*nad2*	J	202/202/200	1224/1224/1215	ATT/ATT/ATT	TAA/TAA/CTG	0/0/0
*trnW*	J	1223/1225/1221	1294/1296/1289			−2/0/5
*trnC*	N	1287/1289/1282	1347/1349/1342			−8/−8/−8
*trnY*	N	1348/1380/1343	1412/1444/1406			−2/1/0
*cox1*	J	1414/1446/1399	2944/2976/2939	?/?/?	T/T/T	1/1/8
*trnL2*	J	2946/2978/2940	3011/3041/3005			1/1/0
*cox2*	J	3034/3042/3007	3696/3728/3691	ATG/ATA/ATA	TAG/TAA/AGT	22/0/1
*trnK*	J	3696/3729/3692	3766/3797/3761			−1/0/0
*trnD*	J	3767/3798/3762	3829/3860/3823			0/0/0
*atp8*	J	3866/3913/3839	4021/4068/4018	ATA/ATT/ATA	TAA/TAA/TAA	36/52/17
*atp6*	J	4015/4062/4012	4689/4736/4686	ATG/ATG/ATG	TAA/TAA/TAA	−6/−7/−7
*cox3*	J	4689/4736/4686	5475/5522/5472	ATG/ATG/ATG	T/T/T	−1/−1/−1
*trnG*	J	5476/5523/5473	5537/5586/5534			0/0/0
*nad3*	J	5538/5587/5535	5891/5940/5888	ATA/ATA/ATA	TAG/TAG/TAG	0/0/0
*trnA*	J	5890/5939/5887	5953/6001/5950			0/0/−2
*trnR*	J	5953/6002/5951	6016/6064/6012			−1/0/0
*trnN*	J	6016/6064/6012	6081/6128/6076			−1/−1/−1
*trnS1*	J	6081/6129/6076	6147/6195/6142			−1/0/−1
*trnE*	J	6148/6196/6143	6210/6259/6205			0/0/0
*trnF*	N	6210/6259/6205	6274/6321/6268			−1/−1/−1
*nad5*	N	6275/6322/6272	7991/8041/7985	ATT/GTG/ATA	T/T/T	0/0/5
*trnH*	N	7992/8042/7986	8056/8104/8049			0/0/0
*nad4*	N	8057/8105/8049	9392/9440/9385	ATG/ATG/ATG	T/T/T	0/0/−1
*nad4L*	N	9386/9434/9379	9676/9721/9651	ATG/GTG/ATC	TAG/TAA/TAA	−7/−7/−7
*trnT*	J	9679/9724/9672	9741/9786/9735			2/2/20
*trnP*	N	9742/9787/9736	9806/9851/9800			0/0/0
*nad6*	J	9808/9853/9805	10,311/10,359/10,305	ATT/ATA/ATC	TAA/TAA/TAA	1/1/4
*cytb*	J	10,311/10,359/10,305	11,450/11,501/11,444	ATG/ATG/ATG	TAG/TAG/TAG	−1/−1/−1
*trnS2*	J	11,449/11,500/11,443	11,516/11,567/11,510			−2/−2/−2
*nad1*	N	11,534/11,585/11,527	12,484/12,535/12,477	TGG/TTG/TTG	TAG/TAG/TAG	17/17/16
*trnL1*	N	12,485/12,536/12,478	12,549/12,600/12,542			0/0/0
*rrnL*	N	12,550/12,601/12,562	13,850/13,894/13,759			0/0/19
*trnV*	N	13,851/13,895/13,845	13,920/13,964/13,914			0/0/85
*rrnS*	N	13,921/13,965/13,916	14,645/14,735/14,538			0/0/1
CR		14,646/14,736/14,539	15,594/15,775/15,587			0/0/0

**Table 3:** Summarized mitogenomic characteristics of the three Chrysochroinae species.

Species		PCGs			rRNAs			tRNAs		A+T-Rich Region		
	Size	A+T	A+T	Size	A+T	A+T	Size	A+T	A+T	Size	A+T	A+T
	(bp)	(%)	Skew	(bp)	(%)	Skew	(bp)	(%)	Skew	(bp)	(%)	Skew
*C. luodiana*	15,594	68.68	0.12	2026	71.92	−0.12	1448	69.96	0.03	949	77.34	0.01
*N. guxngxiensis*	15,755	65.33	0.14	2065	70.46	−0.16	1442	70.39	0.02	1040	76.35	0.08
*Ch. opulenta*	15,587	67.16	0.16	1821	71.83	−0.14	1438	70.51	0.03	1049	78.07	0.06

## Data Availability

The new sequences of complete mitogenomes can be available in NCBI (PP211020, PP133641, PP211021).

## Supplementary Materials

The following supporting information can be downloaded at: https://www.mdpi.com/article/10.3390/genes15101336/s1, Figure S1. The mitogenome maps of *Catoxantha luodiana*. Figure S2. The mitogenome maps of *Nipponobuprestis guangxiensis*. Figure S3. The mitogenome maps of *Chrysochroa opulenta*. Figure S4. The secondary cloverleaf structure for the tRNAs of *Catoxantha luodiana*. Figure S5. The secondary cloverleaf structure for the tRNAs of *Nipponobuprestis guangxiensis*. Figure S6. The secondary cloverleaf structure for the tRNAs of *Chrysochroa opulenta*.
