# Supplementary figures and images for: Three Mitochondrial Genomes of Chrysochroinae (Coleoptera, Buprestidae) and Phylogenetic Analyses
Source: Genes (Basel). 2024 Oct 17;15(10):1336. doi: 10.3390/genes15101336 (PMC11506987; doi:10.3390/genes15101336)

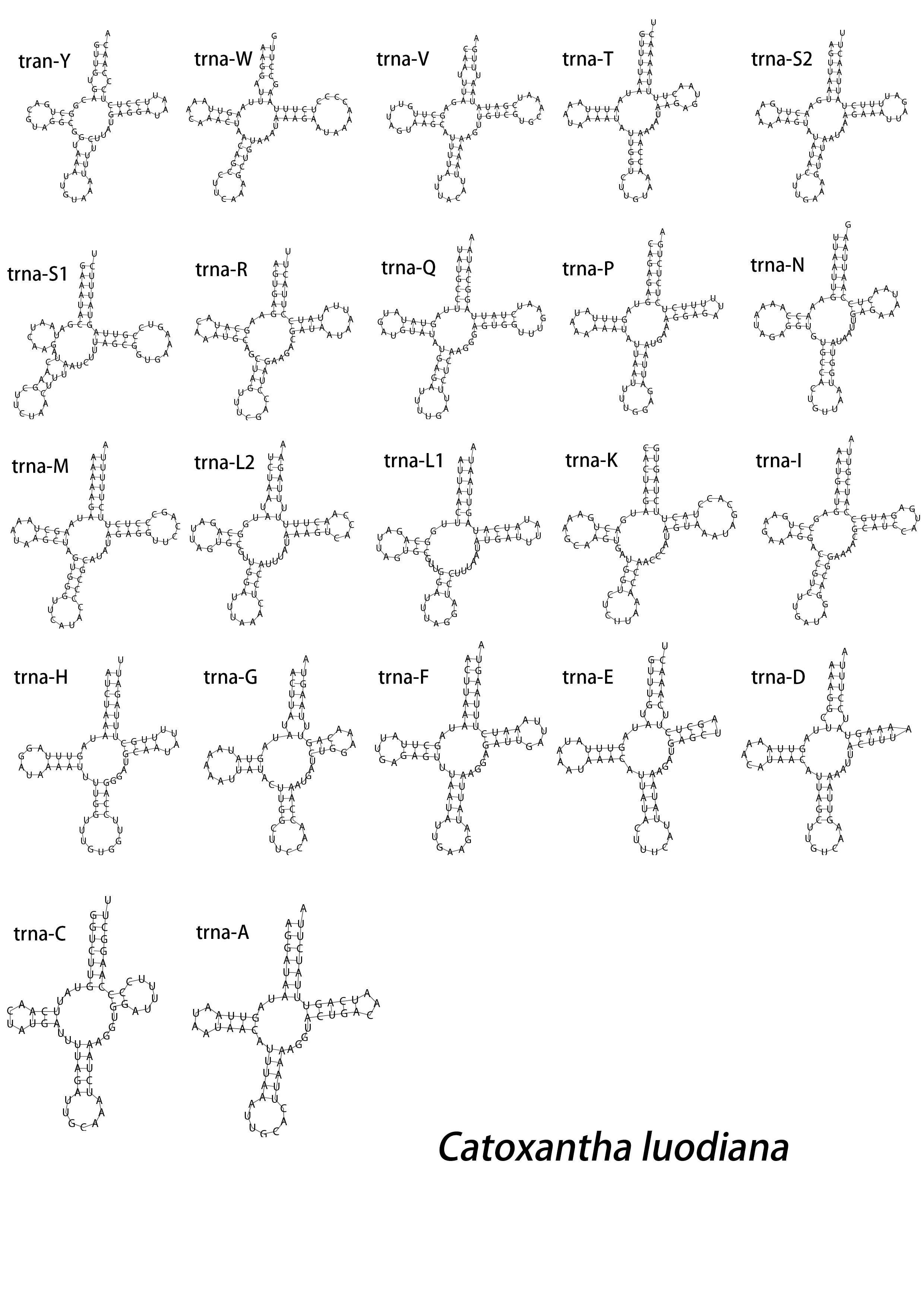

Supplement: Supplementary file 1 [file genes-15-01336-s001.zip › Catoxantha luodiana-tran╗∙╥≥╫Θ.TIF]

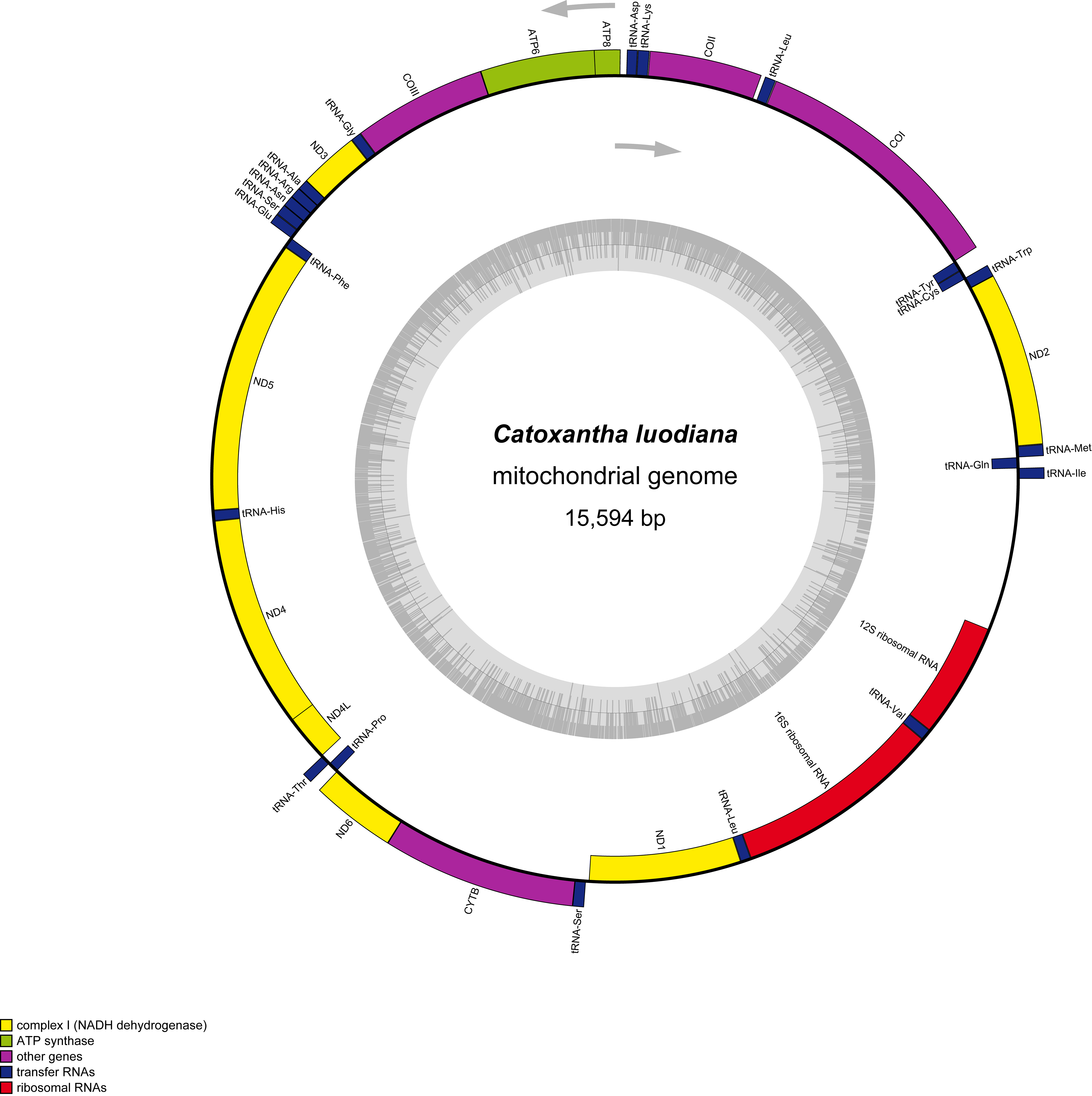

Supplement: Supplementary file 1 [file genes-15-01336-s001.zip › Catoxantha luodiana-╧▀┴ú╠σ╗∙╥≥╫Θ╗╖╫┤═╝╞╫.tif]

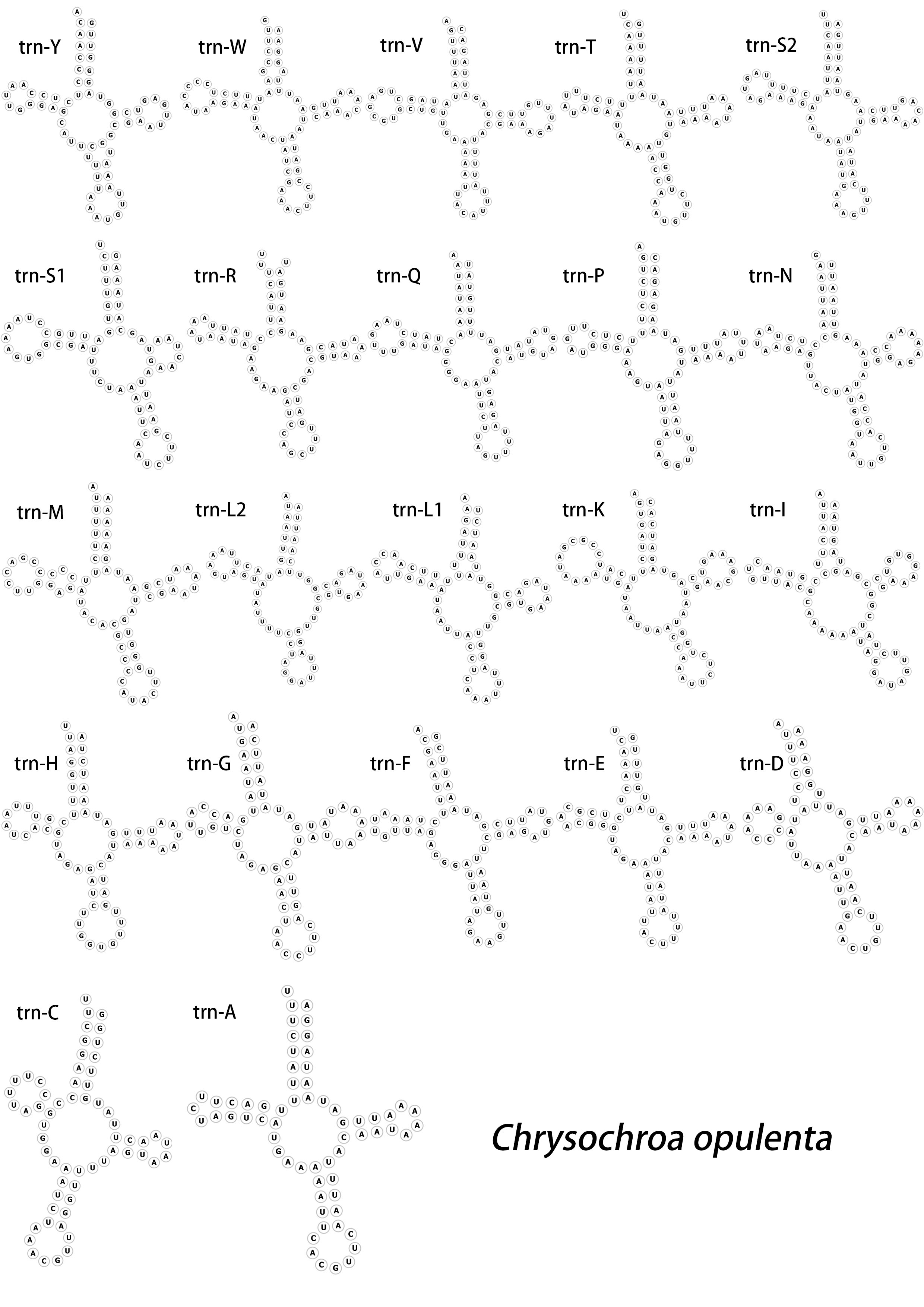

Supplement: Supplementary file 1 [file genes-15-01336-s001.zip › Chrysochroa opulenta-trn╗∙╥≥╫Θ.TIF]

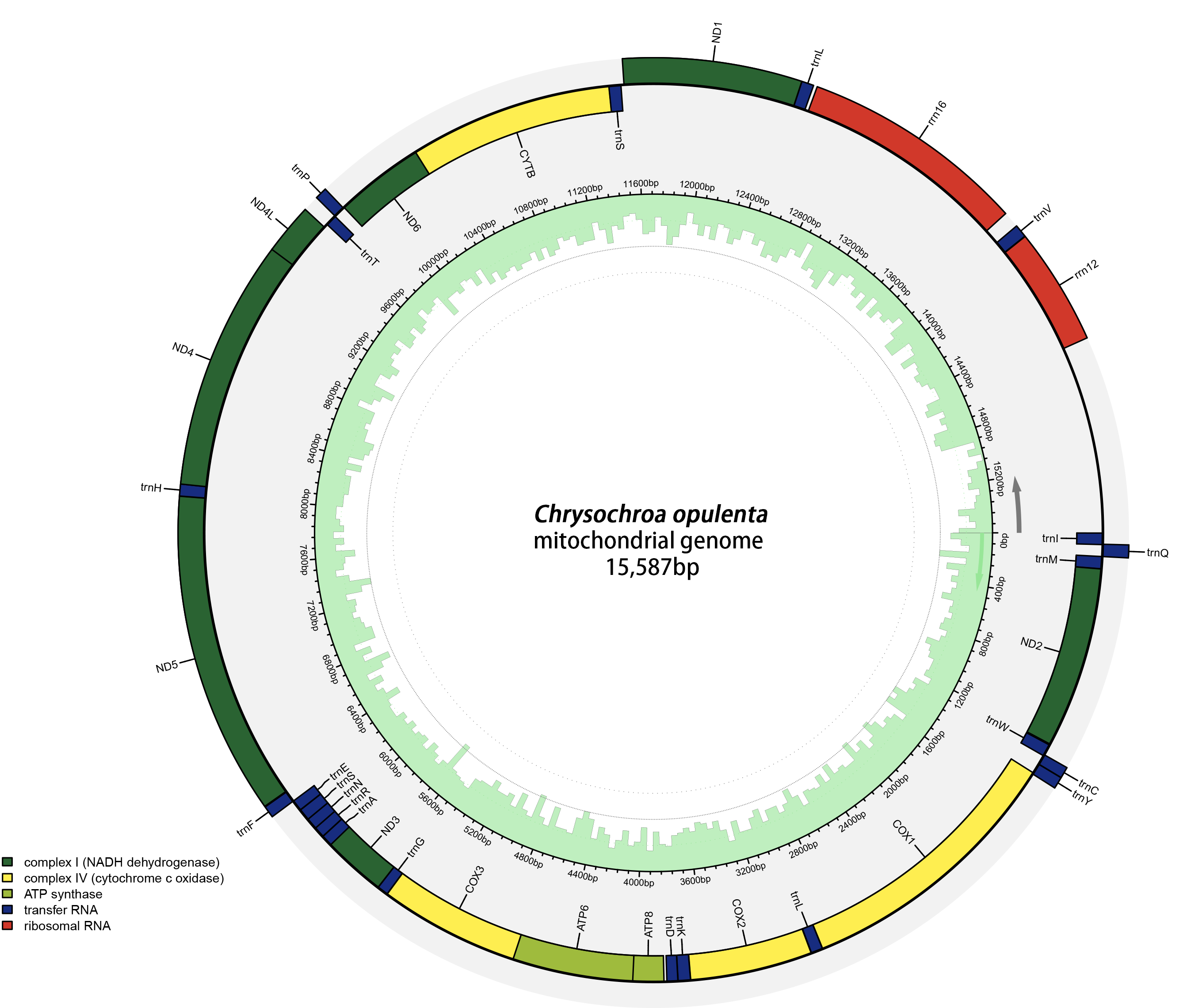

Supplement: Supplementary file 1 [file genes-15-01336-s001.zip › Chrysochroa opulenta-╧▀┴ú╠σ╗∙╥≥╫Θ╗╖╫┤═╝╞╫.tif]

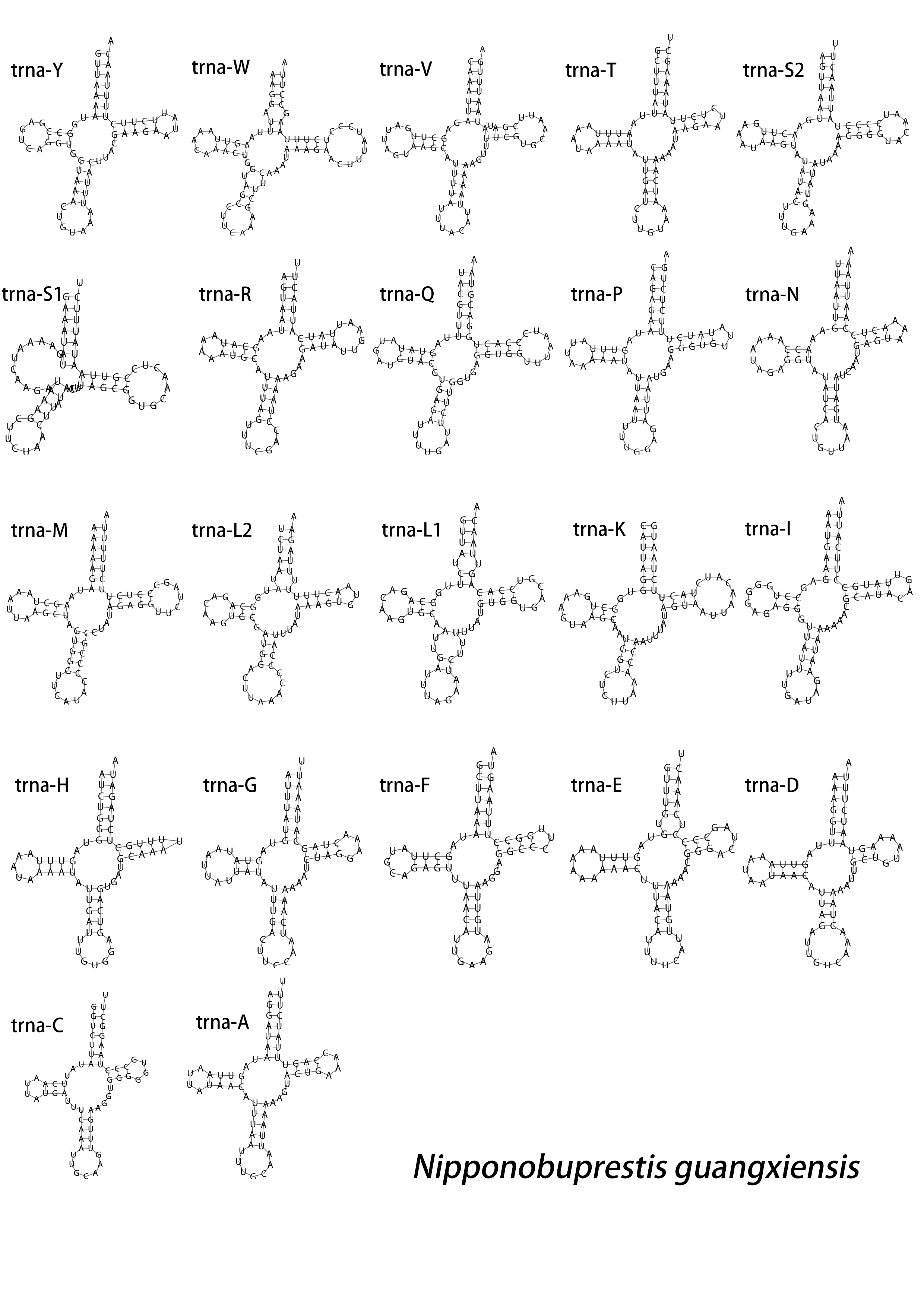

Supplement: Supplementary file 1 [file genes-15-01336-s001.zip › Nipponobuprestis guangxiensis-trna╗∙╥≥╫Θ.tif]

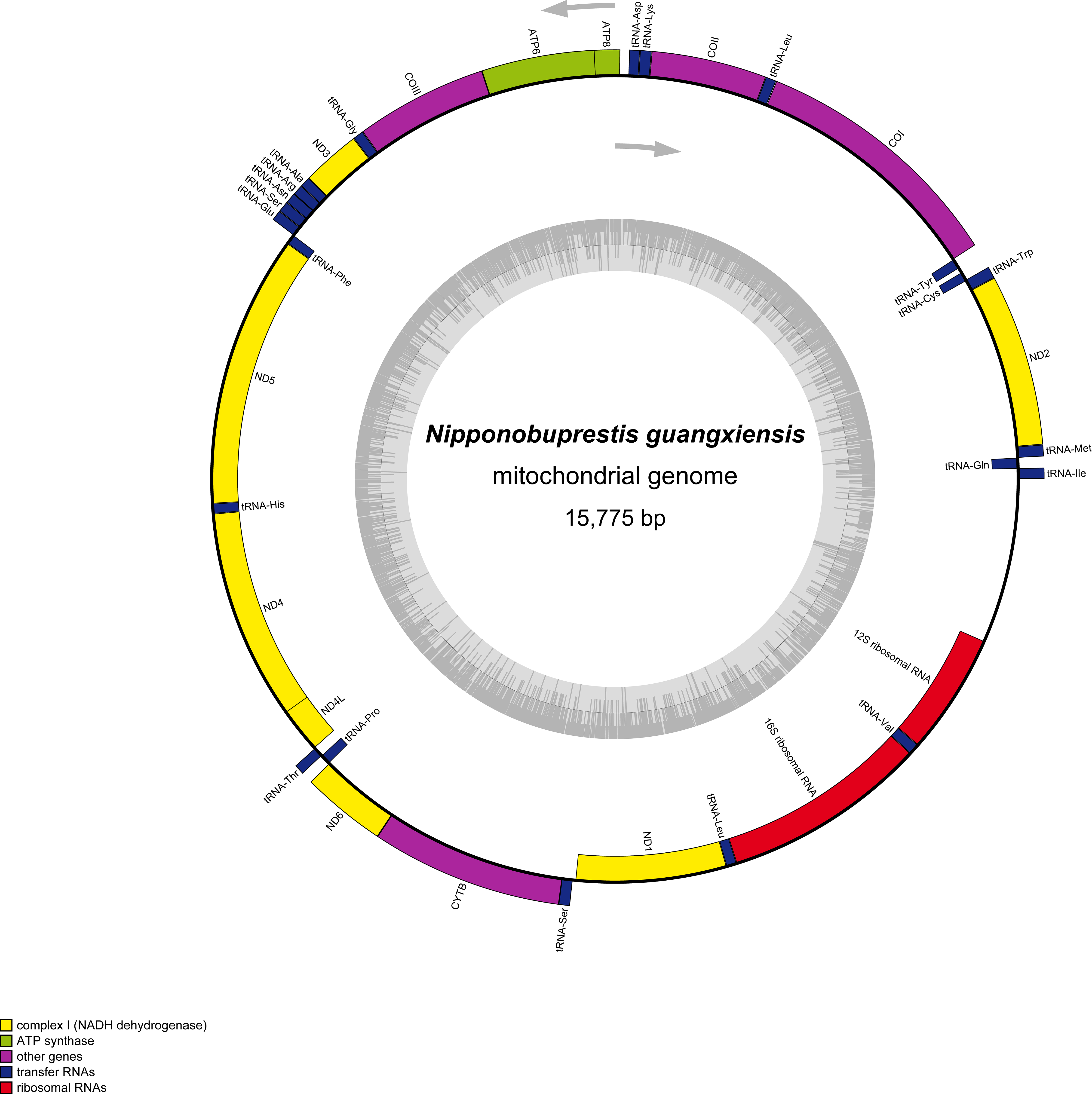

Supplement: Supplementary file 1 [file genes-15-01336-s001.zip › Nipponobuprestis guangxiensis-╧▀┴ú╠σ╗∙╥≥╫Θ╗╖╫┤═╝╞╫.tif]
